# Immunosuppression response to the neonicotinoid insecticide thiacloprid in females and males of the red mason bee *Osmia bicornis* L.

**DOI:** 10.1038/s41598-020-61445-w

**Published:** 2020-03-13

**Authors:** Annely Brandt, Birgitta Hohnheiser, Fabio Sgolastra, Jordi Bosch, Marina Doris Meixner, Ralph Büchler

**Affiliations:** 1 LLH Bee Institute, Erlenstr. 9, 35274 Kirchhain, Germany; 2 Dipartimento di Scienze e Tecnologie Agro-Alimentari, Università di Bologna, Bologna, Italy; 3 CREAF, Bellaterra, 08193 Spain

**Keywords:** Immunology, Physiology, Ecology, Environmental sciences, Agroecology, Animal physiology

## Abstract

Solitary bees are frequently exposed to pesticides, which are considered as one of the main stress factors that may lead to population declines. A strong immune defence is vital for the fitness of bees. However, the immune system can be weakened by environmental factors that may render bees more vulnerable to parasites and pathogens. Here we demonstrate for the first time that field-realistic concentrations of the commonly used neonicotinoid insecticide thiacloprid can severely affect the immunocompetence of *Osmia bicornis*. In detail, males exposed to thiacloprid solutions of 200 and 555 µg/kg showed a reduction in hemocyte density. Moreover, functional aspects of the immune defence - the antimicrobial activity of the hemolymph - were impaired in males. In females, however, only a concentration of 555 µg/kg elicited similar immunosuppressive effects. Although males are smaller than females, they consumed more food solution. This leads to a 2.77 times higher exposure in males, probably explaining the different concentration thresholds observed between the sexes. In contrast to honeybees, dietary exposure to thiacloprid did not affect melanisation or wound healing in *O. bicornis*. Our results demonstrate that neonicotinoid insecticides can negatively affect the immunocompetence of *O. bicornis*, possibly leading to an impaired disease resistance capacity.

## Introduction

Bees (Anthophila) are important pollinators of wild and cultivated plants, and are therefore essential for ecosystem function and agricultural production^1,2^. Social bee species such as honeybees and bumblebees are most known by scientist and the public. However, the majority of the more than 20,000 bee species worldwide are solitary bees^3^. Honeybees form colonies with a single egg-laying queen and thousands of sterile workers that cooperate with nest building, foraging and brood care. Whereas in solitary bee species the female bee builds and provisions her nest and raises her offspring alone, without the cooperation with others^4,5^. Traditionally most pollination service has been attributed to honeybees or bumblebees, but also solitary bee species contribute significantly to the pollination of wild and cultivated plants^1,2^. Worldwide, managed honeybees and bumblebees are utilized to provide pollination service for food crops, but solitary can pollinate some crops more effectively, thereby promoting the fruit set and increasing the overall crop yield^2^.

Over the past decades, serious declines in bee abundance and diversity have been reported in Europe and Northern America^6–8^. Multiple factors, such as habitat degradation, poor nutrition, parasites, pathogens, and pesticides, acting alone or in combination have contributed to these declines. Among pesticides, the use of neonicotinoid insecticides has been highlighted as an important factor underlying bee losses^9–12^. Neonicotinoids act as agonists of the nicotinic acetylcholine receptor, thus disrupting the neuronal cholinergic signal transduction, which results in abnormal behaviour, immobility and death of target pest insects. Beneficial insects such as bees can be exposed through ingestion of contaminated pollen and nectar of treated plants^13–15^.

Harmful sublethal effects of orally ingested neonicotinoids on honeybees (*Apis mellifera* L.)^16^, bumblebees (*Bombus* spp.)^17^, and solitary bees^12,18–21^ have been reported in numerous laboratory and field trials, raising concerns over their widespread use and the potential threat to valuable pollination services for crops and wild plants^6^. So far, the majority of studies have focused on three neonicotinoid insecticides commonly used as seed dressings: imidacloprid, thiamethoxam and clothianidin. These insecticides are now considered to pose a serious risk to bees, and their use in the European Union has been restricted since 2013^22^. However, other neonicotinoids, such as thiacloprid and acetamiprid, are classified as not dangerous for bees, and in some regions or countries are frequently applied to flowering crops when bees are actively foraging^23^. Their potential effects on bees have been largely overlooked, even though thiacloprid and acetamiprid were shown to cause sublethal impairments to bees under agronomically realistic conditions^16,24–26^.

Sublethal exposure to agricultural pesticides has long been acknowledged to have ecologically relevant effects on bees^16,23,27^. However, potential effects on immune function and disease resistance have only recently been recognized^28,29^. Compromised immunity caused by exposure to neonicotinoids has been demonstrated in honeybees^25,26,30^ and bumblebees^28^, but information on solitary bees is largely missing. Honeybee exposure to neonicotinoids has been associated with negatively modulated immune signalling and increased virus replication^30^, with reduced hemocyte numbers, wound healing, and cellular responses to bacterial infection ^25,26,31^, and with reduced activity of an enzyme involved in social immune food sterilisation^32^. In bumblebees, exposure to high doses of imidacloprid reduced constitutive levels of phenoloxidase^28^. Investigating the immune response of other bee taxa is important for three reasons. First, several studies have shown that different bee species may have different sensitivities to pesticides^18,33–38^. Second, different bee species may show differences in routes and levels of pesticide exposure^39,40^. Third, social bees are considered less vulnerable to pesticides, because effects at the individual level can be buffered by the colony (colony resilience^41^). These inter-specific differences have underscored the need to include several bee species in pesticide risk assessment schemes ^18,19,41,42^.

Two *Osmia* species, *Osmia cornuta* and *O. bicornis* have been proposed as model species by the EFSA Guidance Document on the risk assessment of plant protection products on solitary bees^43^. Most bee risk assessment schemes and ecotoxicological studies focus on females^43^. However, assessing the effects of pesticides on males is important for two reasons. First, in the vast majority of bee species, including all solitary species, males are also exposed to pesticides via nectar ingestion. Second, sex-specific differences in humoral immune parameters have been described for two solitary bee species^44,45^. For these reasons, we decided to investigate potential response differences between the sexes.

In this study, we investigate potential differences between the sexes in the immune response of a solitary bee, the red mason bee, *Osmia bicornis* L. (syn. *Osmia rufa* L.) to oral ingestion of thiacloprid. The objectives of our study are: (1) To describe functional parameters (hemocyte density, antimicrobial activity of the hemolymph, and melanisation response) of the immune system; (2) To examine, whether these parameters are affected by orally ingested sublethal concentrations of thiacloprid; (3) To compare the responses of males and females.

## Results

### Food consumption

Bees of both sexes consumed low amounts of sugar solution the first two days of exposure, but uptake increased on day three. Although females are heavier than males (Suppl. Figure 1), they consumed less feeding solution (Table 1). Hence, the total thiacloprid dosage of males in treatment 200 was 2.77 times higher than that of females. Based on these results, additional experiments with adjusted concentrations of 555 µg/kg thiacloprid were performed to reach a similar dosage per mg body weight in females as in males of the treatment 200 group.

**Table 1:** Consumption (mean ± standard error) of sugar solution over three days, thiacloprid uptake per bee and per mg of body weight in males and females. The thiacloprid dosage per body weight of males in treatment 200 is approx. the same as in females in treatment 555 (bold).

Sex	Treatment	Thiacloprid concentration	Sugar solution consumed (mg/bee)	Thiacloprid consumed (µg/bee)	Thiacloprid dosage (ng/mg body weight)
Males	control	0	171.55 ± 15.32	0.000	0.00
	100	100 µg/kg	179.80 ± 23.89	0.018	0.35
	200	200 µg/kg	130.95 ± 25.17	0.026	**0.50**
	555	555 µg/kg	128.45 ± 19.17	0.072	1.34
Females	control	0	97.22 ± 22.3	0.000	0.00
	100	100 µg/kg	79.06 ± 18.42	0.008	0.09
	200	200 µg/kg	80.46 ± 23.39	0.016	0.18
	555	555 µg/kg	85.88 ± 17.55	0.046	**0.52**

Pollen consumption was observed in both males and females. However, it was difficult to quantify because bees spread crumbles of the pollen patty all over the cage.

### Total hemocyte counts

**Figure 1:**
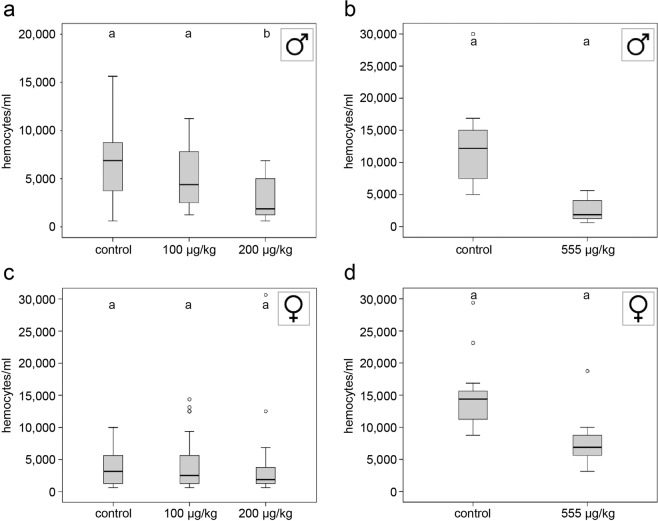
Hemocyte counts in males and females in relation to thiacloprid concentration of feeding solution (**a,b**) Males exposed to 200 µg/kg thiacloprid showed a significantly reduced total number of hemocytes (control, n = 21; 100 µg/kg, n = 23; 200 µg/kg, n = 21; Kruskal-Wallis test, p < 0.0001; post-hoc Mann-Whitney test, control vs. 200 µg/kg, p = 0.001; control vs. 555 µg/kg, p < 0.0001). (**c**) Hemocyte density of females exposed to 100 or 200 µg/kg thiacloprid showed no differences from control (control, n = 32; 100 µg/kg n = 34; 200 µg/kg, n = 34; Kruskal-Wallis test, p = 0.187). (**d**) Exposure to 555 µg/kg resulted in reduced hemocyte density in females (control, n = 18; 555 µg/kg, n = 17; Mann-Whitney test, p = 0.004). Whiskers encompass 95% of the individuals, beyond which outliers (circles reside). Treatments with different letters differ significantly from each other.

In males, a reduction in the total number of hemocytes was observed in the treatment groups 200 and 555 (Fig. 1a, Kruskal-Wallis test, KWT, p = 0.001; Mann-Whitney U test, MWU, control_2016_ vs. treatment 200, p = 0.001; Fig. 1b, KWT, p < 0.001; MWU, control_2018_ vs. treatment 555, p < 0.001, additional statistical information in Table 1). However, in females, the exposure to 100 or 200 µg/kg thiacloprid did not significantly reduce the total hemocyte counts (Fig. 1c, KWT, p = 0.187). Only when we exposed females to 555 µg/kg (comparable to the dosage/mg body weight ingested by males in treatment 200), we observed a significantly reduced number of hemocytes (Fig. 1d, KWT, p < 0.001; *post-hoc* MWU control_2018_ vs. treatment 555, p < 0.001). Unexpectedly, the total number of hemocytes differed between the years (control_2016_ vs. control_2018_: females, MWU, p < 0.001; males, MWU, p = 0.004).

### Antimicrobial activity of the hemolymph

**Figure 2:**
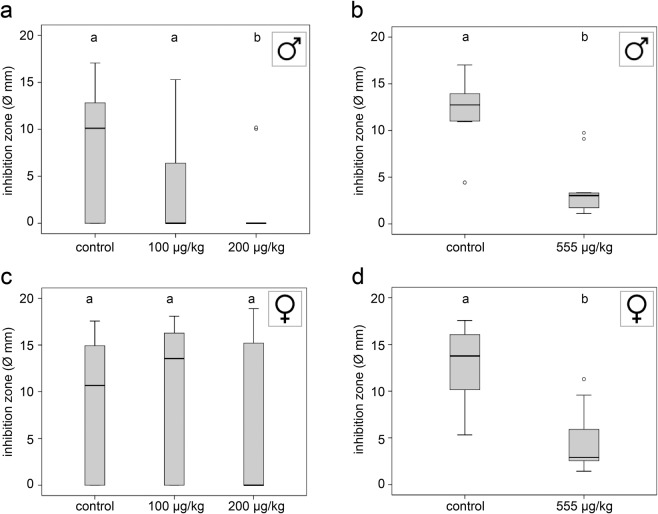
Antimicrobial activity of hemolymph of males and females in relation to thiacloprid concentration of feeding solution. (**a**,**b**) The diameters of inhibition zones were significantly reduced in males exposed to thiacloprid (control, n = 26; 100 µg/kg, n = 19; 200 µg/kg, n = 22; Kruskal-Wallis test, p = 0.002; post-hoc Mann-Whitney test, control vs. 200 µg/kg, p = 0.0008). (**c**) Females exposed to lower concentrations of thiacloprid showed no effect on antimicrobial activity (control, n = 30; 100 µg/kg, n = 25; 200 µg/kg, n = 33; Kruskal-Wallis test, p = 0.454). (**d**) The inhibition zone diameters of the haemolymph of females exposed to the highest concentration (555 µg/kg) were significantly reduced (control, n = 13; 555 µg/kg, n = 16; Mann-Whitney test, p > 0.0001). Whiskers encompass 95% of the individuals, beyond which outliers (circles reside). Treatments with different letters differ significantly from each other.

The size of the inhibition zones was significantly reduced in males exposed to 200 or 555 µg/kg thiacloprid (Fig. 2a,b; KWT, p = 0.002; MWU control_2016_ vs. treatment 200, p = 0.0008; control_2018_ vs. treatment 555, p < 0.001; additional statistical information in Table 1). The antimicrobial activity was not significantly reduced in females exposed to 100 or 200 µg/kg thiacloprid (Fig. 2c; KWT, p = 0.454). Only females exposed to 555 µg/kg thiacloprid showed significantly reduced inhibition zones (Fig. 2d, MWU, control_2018_ vs. treatment 555, p < 0.001).

In some individuals, the injection procedure obviously caused some damage, especially males where affected, probably because the handling of the smaller males is more difficult. In some bees, the movement of the hindlegs where impaired, some died. These affected individuals were censored and excluded from the measurements (females: control = 17%; treatment 100 = 11%; treatment 200 = 16%; males: control = 32%; treatment 100 = 31%; treatment 200 = 20%). There was no statistically significant difference between the treatment groups.

We tested the effect different experimenters have on the variance of the inhibition zone diameters using Lysozyme as a standard. Interestingly, we found a significant statistical difference between the measurements of inhibition zone diameters done by two persons (Suppl. Table 2). Therefore, only a single person pipetted the hemolymph and measured the inhibition zone to increase the technical repeatability of the assay.

### Encapsulation response

**Figure 3:**
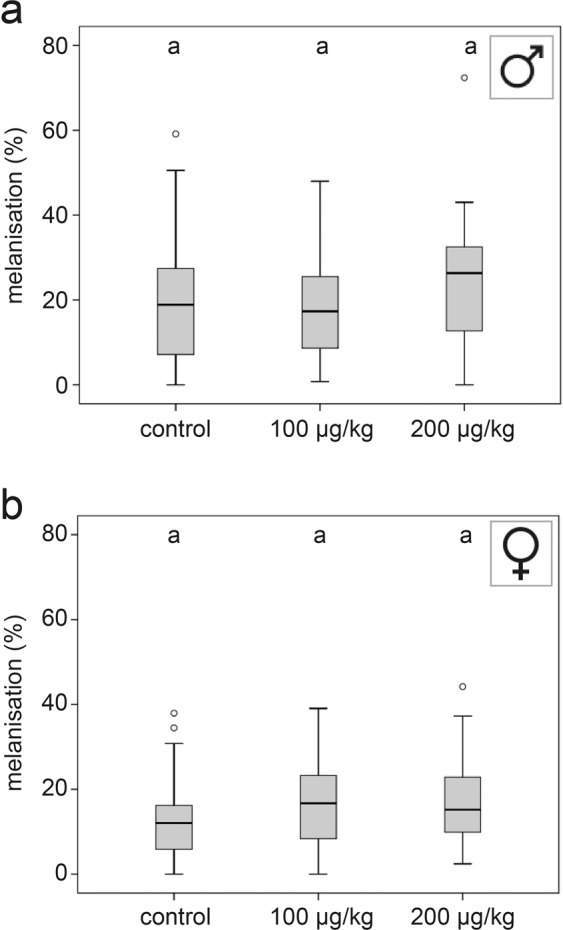
Exposure to thiacloprid had no effect on the melanisation response. The three day-exposure of young males (**a**; control, n = 22; 100 µg/kg, n = 26; 200 µg/kg, n = 27; Kruskal-Wallis test, p = 0.295), and females (**b**) had no effect on the melanisation of an implanted nylon filament compared to control (control, n = 22; 100 µg/kg, n = 26; 200 µg/kg, n = 27; Kruskal-Wallis test, p = 0.303). Whiskers encompass 95% of the individuals, beyond which outliers (circles reside). Treatments with different letters differ significantly from each other.

The encapsulation response to thiacloprid exposure was not significantly reduced in males (Fig. 3a; KWT, p = 0.295) or females (Fig. 3b; KWT, p = 0.303; additional statistical information in Table 1).

## Discussion

We demonstrate that the commonly used neonicotinoid insecticide thiacloprid impairs the immune defence of a solitary bee, *Osmia bicornis*. The number of hemocytes, as well as the antimicrobial activity of the hemolymph, were negatively affected by sublethal, environmentally relevant concentrations of thiacloprid. Although males are smaller than females, they consumed significantly more food than females. This leads to higher exposure in males even at field-realistic concentrations of thiacloprid, probably explaining the different concentration thresholds observed between the sexes. In contrast to honeybees, dietary exposure to thiacloprid did not affect the melanisation or wound healing response in *O. bicornis*.

Thiacloprid is generally regarded as non-harmful to bees based on its relatively low acute toxicity to honeybees (oral acute LD50_48h_ = 17.32 μg/honeybee^46^). The thiacloprid concentrations we used fall within the range of concentrations found in field situations. In pollen collected by honeybees, mean thiacloprid residues were 75.1 µg/kg (max.: 1002 µg/kg, mean prevalence of 17.7%) and 6.5 µg/kg in honey (max.: 208 µg/kg, 64% prevalence^47^). In the German bee monitoring project, the highest concentration of thiacloprid in beebread samples was 498 µg/kg^48^.

In general, hemocytes are the key components of the cellular immune defence of insects. They are responsible for phagocytosis or encapsulation of pathogens as well as for wound closure^49,50^. On average, female *O. bicornis* had lower total hemocyte counts than males. Interestingly, only the highest concentration of 555 µg/kg thiacloprid reduced the hemocyte density in females. In contrast, we found a reduction of hemocyte number in males dosed as low as with 200 µg/kg, indicating a disturbance of the immune system after exposure to concentrations frequently found under field conditions^48^. This reduction of hemocytes in neonicotinoid-exposed *O. bicornis* could limit the capacity of the red mason bees to mount a rapid immune response^51^.

One essential functional aspect of the bee’s immune system is the induced immunity as measured by the humoral antimicrobial activity of the hemolymph. The humoral immune response is mediated by antimicrobial peptides that are produced by hemocytes and fat body cells. These are biologically active molecules with antibacterial, antifungal, or antiviral properties^50,52^. Together with the reduction of hemocyte density, the reduced antimicrobial activity in thiacloprid-exposed males (200 and 555 µg/kg) and females (555 µg/kg) could likely impair the immune strength of *O. bicornis* and increase their susceptibility towards pathogens.

In insects, hemocytes actively encapsulate infected cells or intruding pathogens and migrate to wounds in order to close them. Concurrently, hemocytes produce prophenoloxidase, the precursor of the enzyme phenoloxidase, which catalyses the melanisation reaction^50^. In both female and male *O. bicorni*s, a melanisation reaction of an implanted nylon fibre was observed. However, contrary to findings in *A. mellifera* workers^26^ and queens^25^, where thiacloprid significantly reduced the melanisation response, no reduction was observed in *O. bicornis*. This finding may reflect differences in the pathogen and parasite pressure of these two species. In contrast to *O. bicornis*, honeybees have co-evolved with hemolymph sucking parasites (*Acarapis woodi*, *Tropilaelaps* spp., and recently *Varroa destructor*) which might explain this divergence between *O. bicornis* and *A. mellifera*. Our results emphasise the need to test different species and not to generalize the findings of honeybee studies to other bee species.

Solitary bees have evolved under a different selective pressure than social bees like honey bees or bumble bees, which have evolved specific behavioural, physiological, and organizational adaptations to combat the increased risk of disease inside a colony^51,53^. Hence, the variation in pathogen-specific selective pressure may result in immunocompetence dissimilarities across species.

A comparative analysis about the immune system and the effects of pesticide stressors on immune strength of bee species is difficult since comprehensive information is even missing for honeybees and much more for solitary bees. Genomic analysis of honeybees shows that the basic set of molecules defining the insect immune system is present. However, compared to the sequenced *Drosophila* and *Anopheles* genomes, honeybees possess only about one-third as many genes implicated in insect immunity^50^. This has been related to the eusociality of honeybees. It has been hypothesised that honey bees do not need a more diverse immune repertoire since they are confronted to a less diverse, co-evolved, set of pathogens and that social defence mechanisms, like grooming or brood hygiene lessen the pathogen pressure^50^. Since solitary bee species have no social defence mechanisms, it has been expected that they have a more diverse immune repertoire than eusocial bees^44^. Strachecka *et al*. investigated the chemical defence mechanisms of red mason bees. Indeed, *O. bicornis* had generally higher activities and concentrations of defence compounds in the hemolymph than eusocial bees^44^. However, since the genomes of solitary bee species have not yet been sequenced, it is not possible to compare the genes implicated in immunity to further substantiate this hypothesis.

Neonicotinoids have been shown to affect the immunocompetence of honeybees. Like in *O. bicornis*, thiacloprid has been shown to reduce the hemocyte density, especially the proportion of active, differentiated immune cells in honeybees as well as the antimicrobial activity of the hemolymph^25,26^. Neonicotinoids negatively modulate NF-κB immune signalling and promote the replication of deformed wing virus^30^. Moreover, thiacloprid increases the mortality of *Nosema ceranae* infected honeybees^54^. Whether neonicotinoids affect the disease susceptibility of *O. bicornis* remains to be elucidated.

The observed differences in immunocompetence between male and female *O. bicornis* are likely related to the differences in food uptake, resulting in unequal exposure levels. Indeed, we observed huge differences in absolute food uptake between females and males across all four treatments. For example, males of the 200 treatment consumed 0.5 ng thiacloprid per mg body weight, whereas females consumed only 0.18 ng/mg. After including an additional higher concentration of 555 µg/kg, to account for these differences, reduction of immunocompetence was also observed in females.

The higher food consumption of males can be explained by behavioural differences between the sexes in the post-emergence phase. On average, males emerge 2–4 days before females. They actively patrol nesting sites and try to mate with emerging females, most of which are mated right upon emergence. Once mated, newly emerged females typically hide in crevices and undergo a 2–5-day pre-nesting period, during which they complete ovary maturation^5^. We observed similar behavioural patterns in our experimental flight cages where males were flying and walking extensively, whereas females were mostly hiding in the artificial nesting tubes.

Interestingly, sex-specific differences in immune parameters of *O. bicornis*^44^ and *Megachile rotundata*^45^ were previously reported, indicating lower concentrations and activity levels of phenoloxidase and alkaline phosphatase, and of enzymatic and non-enzymatic antioxidant and proteolytic systems in males. Based on our experimental data, we cannot determine whether physiological or behavioural differences that result in different exposure levels or both caused the divergent immunosuppressive effects between the sexes.

The current risk assessment scheme for the approval and authorization of pesticides focuses on *A. mellifera*, implicating that data obtained with honey bees can be extrapolated to other bee species without considering inter-specific differences^18^. *A. mellifera* is generally considered as extremely sensitive to pesticides and a good indicator species for environmental pollution^55,56^. However, the extrapolation of a risk assessment to other bees may not be appropriate. The response to pesticides and the differences in the level of exposure may vary significantly between bee taxa^38,43^ making it difficult to predict which bee species are more or less susceptible to pesticides^15^. Indeed, our results indicate clear differences between red mason bees and honeybees and emphasize the need for comparative studies and independent pesticide risk assessment procedures for non-*Apis* bees.

Currently, only the food intake of adult females is considered as relevant; while food intake of adult male is regarded “not relevant” or unknown^43,57^. However, our results show clear sex-specific differences in food uptake and exposure level and – most likely as a consequence - an elevated response of males towards field realistic thiacloprid concentrations. Therefore, male bees should be included in future risk assessment schemes for approval and authorization of pesticides.

The interaction of pathogens and pesticides is of grave concern in relation to pollinator health. Multiple stressors on bee health act not in isolation, but rather interact, with possible synergistic effects on bees and bee populations^8,58^. Our findings highlight the importance of including non-*Apis* species and both sexes in eco-toxicological studies and risk assessment procedures^27^. This has also a strong economic and ecological relevance since many bee species, other than *A. mellifera*, contribute decisively to crop and wild plant pollination^2^.

## Methods

### *Osmia bicornis* population management

*Osmia bicornis* cocoons reared at the arboretum of the Botanical Garden of the University of Rostock (Germany) were obtained from Johann-Christoph Kornmilch (bienenhotel.de, Rostock, Germany). The cocoons were shipped in January and stored in the dark at 4°–7 °C. To induce adult emergence, cocoons were placed in cages (30 × 30 × 30 cm, Aerarium, bioform, Nürnberg, Germany) under natural light at room temperature (1^st^ of March–4^th^ of May 2016 and 2018; average temperature 22.6 °C, min 20.0 °C, max. 24.2 °C; relative humidity 62%, min. 58.8%, max. 72%, datalogger EL-USB-2, Lascar electronics, Whiteparish). We separated female and male cocoons based on size, and kept them in separate cages for the whole study. Approximately 24 hours after emergence, we transferred the bees to new cages in groups of 5 to 10 individuals, where the exposure to thiacloprid took place. We conducted three independent replicates (cages) per treatment and sex for each immune assay.

### Test solutions

A stock solution of 2 mg/ml thiacloprid (Sigma Aldrich, St. Louis, USA; purity 99.9%) in acetone was prepared in a glass flask and stored in the dark at 15 °C until use. The stock solution was added to a 50% feeding solution of invert sugar (Ambrosia, Germany) in distilled water (w/v) to reach three test concentrations: 100, 200, and 555 µg/kg (henceforward referred to as treatment 100, 200 and 555). The final concentration of acetone in the test solutions was adjusted to 0.0086% (v:v) in all treatments.

A pollen patty was prepared from pollen collected by honey bees at the Bee Institute Kirchhain or obtained from Imkereibedarf Bährle (Aschaffenburg, Germany). The pollen was stored at −20 °C for 8–10 months and then pulverized in a coffee grinder. The resulting pollen powder was mixed with the thiacloprid-spiked feeding solution to obtain the same concentrations as the feeding solution (100, 200, 555 µg of thiacloprid per kg of pollen patty).

### Neonicotinoid exposure

Test cages were placed close to each other (~2 cm), so bees of both sexes could see, hear and smell each other. Fragments of egg carton and 5 cm segments of black plastic drinking straws were placed in each flight cage as hiding sites. Cages were kept at room temperature under natural light conditions.

Caps of 1.5 ml microcentrifuge tubes (Eppendorf, Hamburg, Germany) were cut off and used as containers for the feeding solution (two caps per cage) and pollen paste (two caps per cage). The bottoms of the caps were coloured red or blue and placed on a green piece of cardboard to attract the bees. Both females and males found the food sources immediately, and feeding was observed frequently.

Each cage was assigned to one of four thiacloprid treatments and exposed for three days. Feeding caps were weighed and renewed every day. For each treatment group and sex, three cages of 6 to 10 individuals were set up on independent dates. In 2016, we tested the 0, 100 and 200 µg/kg treatments (Table 2). Then, based on the discrepancies observed between the dosage per body weight in males and females, additional experiments with 0 and 555 treatments were conducted (three groups for each treatment and sex on independent dates in 2018) to obtain data on a dosage taken up by females equivalent to the 200 treatment in males (Table 2). In order to minimize the variance in the following functional immune assays caused by different experimenters, only one person at a time analysed the three treatment groups (Suppl. Tables 1 and 2).

**Table 2:** Results of immune test and number of individuals per treatment group and sex for each experiment, at least three independent test runs were conducted. The total number of males and females (*n*) included in data analysis is given for each experiment (s.e.m = standard error of the means).

Immune parameter	Experiments in 2016			Experiments in 2018	
mean ± s.e.m, *n* = number of individuals	*Control*	*100 µg/kg*	*200 µg/kg*	*Control*	*555 µg/kg*
**Males**					
Total hemocyte count (cells/ml)	6815.48 ± 782.81	5135.87 ± 596.54	2946.43 ± 437.70	12109.38 ± 1496.53	2708.33 ± 699.29
*n*	21	23	21	16	15
Inhibition zone (mm)	7.02 ± 2.88	3.59 ± 1.04	0.92 ± 1.39	12.42 ± 3.10	3.33 ± 0.86
*n*	26	19	22	16	15
Censored individuals					
Encapsulation (% grey value)	20.26 ± 3.42	18.66 ± 2.46	24.39 ± 2.94	—	—
	22	26	27		
**Females**					
Total hemocyte count (cells/ml)	3574.22 ± 457.41	4264.71 ± 691.95	3382.35 ± 916.22	14791.67 ± 1121.91	8198.53 ± 1010.70
*n*	32	34	34	18	17
Inhbition zone (mm)	8.12 ± 1.23	9.48 ± 1.56	6.81 ± 1.39	13.6 ± 3.62	4.33 ± 1.08
*n*	30	25	33	13	16
Encapsulation (% grey value)	13.44 ± 1.86	16.44 ± 1.98	17.37 ± 1.91	—	—
*n*	22	26	27		

### Hemolymph extraction and total hemocyte counts

Bees were anesthetized on ice, and hemolymph was extracted by inserting a microinjection needle (Hartenstein, Würzburg, Germany) into the proximal abdomen between the 3^rd^ and 4^th^ tergum^25,26^. Hemolymph (1 µl) was transferred to PCR-tubes (Biozym, Hessisch Oldendorf, Germany) containing 1 µl of DAPI-staining solution (4’,6-diamidino-2-phenylindole; 1:100 dilution, lifetechnologies, Carlsbad, California, USA) and 3 µl PBS (pH 7.4; Sigma Aldrich, St. Louis, USA). Hemocytes were counted in a counting chamber (Bürker, Carl Roth, Karlsruhe, Germany) under a phase contrast/fluorescent microscope (Leica DMIL, Leica camera DFC 420 C)^25,26^.

### Encapsulation response

A 2.5 mm nylon filament was partly inserted into the abdomen of anesthetized bees as previously described for honeybees^25,26^. After implantation, females were transferred to 1.5 ml microcentrifuge tubes and males to PCR-tubes with holes poked through the cap and sidewalls. After four hours at room temperature, the nylon filament was extracted, fixed in formaldehyde solution for at least 1 hour, rinsed three times in PBS, and subsequently mounted in glycerol (85%, Carl Roth). For each explant, three pictures were taken at different focal depths. The mean grey value per filament was calculated using image analysis software and taken as a measure of melanisation^59^. The mean grey value of a non-implanted filament was subtracted from the mean grey value of the implanted filaments^25^.

### Antimicrobial response

On day two of the exposure phase, the immune system was challenged by the injection of 1 µl of heat-inactivated *Escherichia coli* (OD 0.5)^25,26^. Hemolymph was collected and stored at −20 °C. Bacterial test plates (ø 9 cm) were prepared by adding 0.8 ml of live *Micrococcus luteus* bacteria suspension (OD 0.5) to 150 ml of sterile broth medium (48 °C, 1.5 g Agar No. 1, Oxoid; 3.75 g nutrient broth, Applichem). For each test plate, five holes (ø 3.33 mm) were punched into the agar with a 1 ml pipette tip, and 1 µl of hemolymph solution was added to each hole. Plates were incubated at 38 °C overnight, and the diameter of inhibition zones was measured with a digital calliper^25,26^.

### Repeatability of measurements

To investigate possible sources of variance and estimate the technical repeatability of our functional measurements of hemocyte density, antimicrobial activity of the hemolymph, and melanisation response, we conducted additional experiments. For these experiments, we collected adult *Apis mellifera* workers from an apparently healthy colony that was not treated against *Varroa destructor* (December 18, 2019). For hemocyte counts, we extracted hemolymph as described before^26^. Two experimenters (for details, see Suppl. Table 1) counted the hemocytes of 30 bees independently. Since hemocytes are mobile, we counted the cells as fast as possible.

For the melanisation response, we implanted and prepared 30 transparent nylon fibres as described before^26^. A single experimenter (Suppl. Table 1) made the microscopic pictures of the same implants twice and conducted the subsequent image analysis independently from each other. To reduce the technical variance, we led the microscope lamp warm up for 30 min before we started to take the pictures.

To estimate for the methodological repeatability of inhibition zone assays, we applied 1 µl of a standard solution of lysozyme (1 mg/1 ml, Applichem, Darmstadt, Germany) on *M. flavus* inoculated petri dishes. To cover different technical sources of variance, we tested three scenarios (a) pipetting done by two persons, (b) measuring of the inhibition zone diameters done by two persons, (c) repeated measurements of the inhibition zone diameters done by a single person.

### Statistical methods

All statistical tests were run with SPSS for Windows (v. 20). Total hemocyte counts, melanisation/mean grey values, and mean diameters of inhibition zones were not normally distributed. Thus, non-parametric statistics were applied^25,26^. Each immunocompetence measure was compared across treatments using Kruskal–Wallis tests (KWT) followed by *post-hoc* pair-wise comparisons with Mann–Whitney U tests (MWU) and Bonferroni corrections (when more than two groups were compared)^25,26^. In order to calculate the repeatability of the functional measurements, we first applied a one-way ANOVA and subsequently calculated the approximate repeatability values from the F ratio and mean squares among groups/mean squares within groups according to Lessells and Boag^60^.

### Ethics

Ethical approval and the licences were obtained from the Hessian Regional Council of Giessen (RPGI), Germany.

## Supplementary information

Supplementary information.

## Data Availability

The datasets supporting this article have been uploaded as part of the supplementary material.
