# Supplementary material for: Immunosuppression response to the neonicotinoid insecticide thiacloprid in females and males of the red mason bee Osmia bicornis L
Source: Sci Rep. 2020 Mar 13;10:4670. doi: 10.1038/s41598-020-61445-w (PMC7070012; doi:10.1038/s41598-020-61445-w)
Supplement: Supplementary file 1 — Supplementary information. [file 41598_2020_61445_MOESM1_ESM.pdf]

Immunosuppression response to the neonicotinoid insecticide thiacloprid in females and males of the red mason bee *Osmia bicornis* L.

Annely Brandt[1*], Birgitta Hohnheiser[1], Fabio Sgolastra[2], Jordi Bosch[3], Marina Doris Meixner[1], Ralph Büchler[1]

[1]LLH Bee Institute, Erlenstr. 9, 35274 Kirchhain, Germany;

[2]Dipartimento di Scienze e Tecnologie Agro-Alimentari, Università di Bologna, Bologna, Italy;

[3]CREAF, Bellaterra 08193, Spain

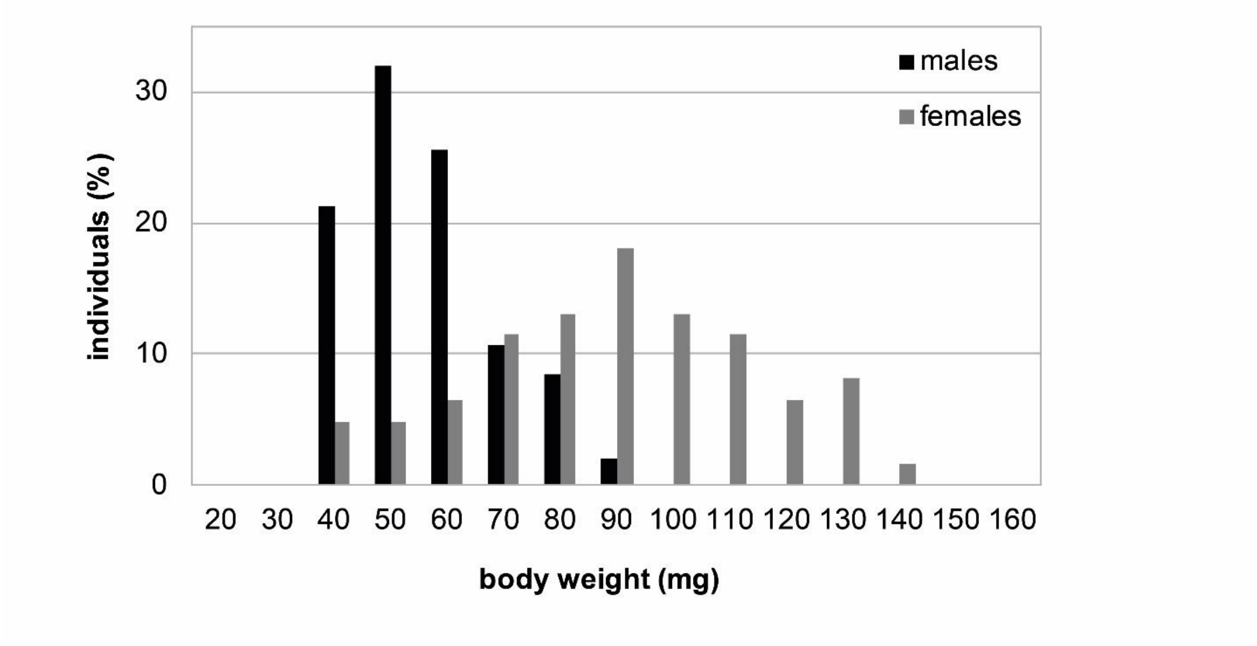

Suppl. Fig. 1 Body weight of individual bees. The weight of individual males (n = 269) and females (n = 327) was measured after the immune tests.

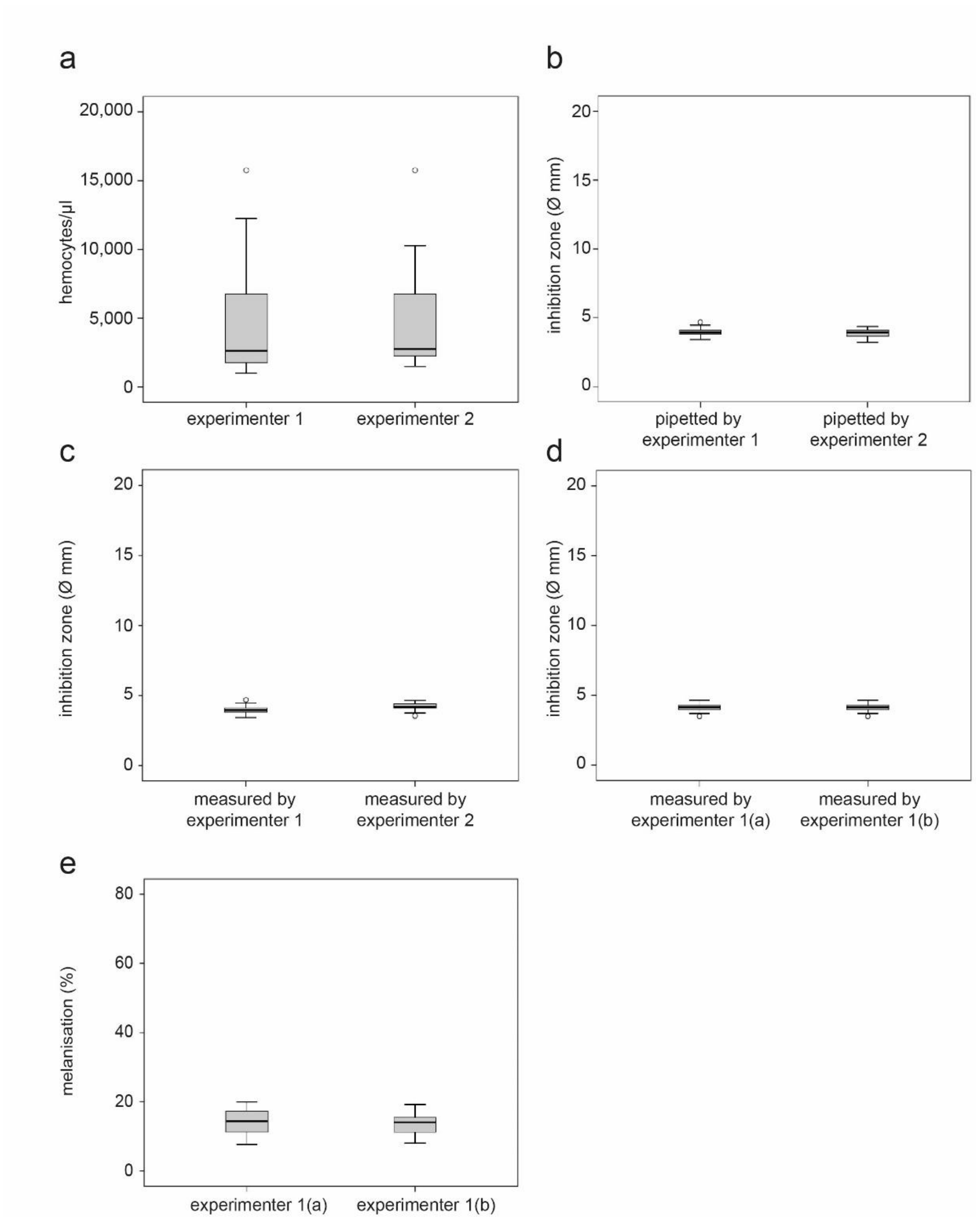

Suppl. Fig. 2 Methodological repeatability of functional measurements of bee immunity. (**a**) The hemocytes of individual *Apis mellifera* worker bee (n = 30) were counted independently by two experimenters (1 and 2). (**b**) The inhibition zones of 1 µl of a standard lysozyme suspension (1mg /1 ml) were pipetted on the *M. flavus* inoculated pertri dishes by 2 experimenters (n = 30 per person), the next day the inhibition zone diameter (mm) was measured with a electronic caliper by experimenter 1. (**c**) The inhibition zones of 1 µl of a standard lysozyme suspension was pipetted by experimenter 1 (n = 30) and measured with a electric caliper independently by experimenters 1 an 2. (**d**) The inhibition zones of 1 µl of a standard lysozyme suspension was pipetted by experimenter 1 (n = 30) and measured twice with an electric caliper by experimenter 1. (**e**) Microscopic pictures of the implanted nylon fibres of *A. mellifera* worker bees were taken independently by experimenter 1 and 2. Whiskers encompass 95% of the individuals, beyond which outliers (circles reside).

Supplemental Table 1 **Experiments were conducted by different persons**

**Assays**	**2016**	**2018**	**2019**
hemocyte counts	BH	AB	AB/BB
melanisation assays	BH	AB	AB/AB
inhibition zone assays	BH	AB	AB/AB/DB
hemolymph extraction	SB	SB	AB

BH	Birgitta Hohnheiser
AB	Annely Brandt
BB	Bastian Barthelmess
DB	Dominique Brandt
SB	Sandra Backhaus

Supplemental Table 2. One-way analysis of variance and calculation of repeatability

source of variation	n	df	Sum of squares	Mean squares	*F* ratio	p-value	repeatability (r)
**hemocyte counts**							
among group		1	292252.604	292252.604	0.024	0.877	-0.034
within group	30		705432942.708	12162636.943			
**inhibition zone assay (a) pipetted by 2 persons, measured by 1 person**							
among group		1	0.78	0.078	0.935	0.338	0.25
within group	30	58	4.826	0.083			
**inhibition zone assay (b) pipetted by 1 person, measured by 2 persons**							
among group		1	0.835	0.835	10.985	0.002	0.013
within group	60	58	4.411	0.076			
**inhibition zone assay (b) pipetted by 1 person, measured twice by 1 person**							
among group	30	1	0.000	0.000	0.006	0.937	-0.034
within group		58	3.918	0.068			
**melanisation assay**							
among group		1	0.917	0.917	0.090	0.765	-0.493
within group	30	58	590.21	101.756			
